# New algorithms to compute the nearness symmetric solution of the matrix equation

**DOI:** 10.1186/s40064-016-2416-x

**Published:** 2016-07-07

**Authors:** Zhen-yun Peng, Yang-zhi Fang, Xian-wei Xiao, Dan-dan Du

**Affiliations:** School of Mathematics and Computing Science, Guangxi Colleges and Universities Key Laboratory of Data Analysis and Computation, Guilin University of Electronic Technology, Guilin, 541004 Guangxi China

**Keywords:** Symmetric matrix, Matrix equation, Iteration method, AVMM method, Matrix nearness problem, 15A24, 15A39, 65F30

## Abstract

In this paper we consider the nearness symmetric solution of the matrix equation *AXB* = *C* to a given matrix $\tilde{X}$ in the sense of the Frobenius norm. By discussing equivalent form of the considered problem, we derive some necessary and sufficient conditions for the matrix $X^{*}$ is a solution of the considered problem. Based on the idea of the alternating variable minimization with multiplier method, we propose two iterative methods to compute the solution of the considered problem, and analyze the global convergence results of the proposed algorithms. Numerical results illustrate the proposed methods are more effective than the existing two methods proposed in Peng et al. (Appl Math Comput 160:763–777, 2005) and Peng (Int J Comput Math 87: 1820–1830, 2010).

## Background

The well-known linear matrix equation

1 $$AXB = C,$$

has been considered by many authors. Such as the generalized singular value decomposition method to compute the symmetric solutions, the reflexive and anti-reflexive solutions, the generalized reflexive solutions and the least-squares symmetric positive semidefinite solutions were studied by Chu (1989) (see also Dai 1990), Peng et al. (2007), Yuan et al. (2008) and Liao et al. (2003), respectively. The quotient singular value decomposition method to compute the least squares symmetric, skewsymmetric and positive semidefinite solutions were studied by Deng et al. (2003). The generalized inverse method to compute the reflexive solutions, the asymmetric positive solutions and the Hermitian part nonnegative definite solutions were considered by Cvetkovic-iliic (2006), Arias et al. (2010) and Dragana et al. (2008), respectively. The matrix-form CGNE (Bjorck 2006) iteration method to compute the symmetric solutions, the skew-symmetric solutions and the least-squares symmetric solution were given by Peng et al. (2005), Huang et al. (2008) and Lei et al. (2007) (see also Peng 2005), respectively. The matrix-form LSQR iteration method to compute the least-squares symmetric and anti-symmetric solutions were given by Qiu et al. (2007). The matrix-form BiCOR, CORS and GPBiCG iteration methods and the matrix-form CGNE iteration method to solve the extension from of the matrix Eq. (1) were studied by Hajarian (2015a, b) and Dehghan et al. (2010), respectively.

The problem of finding a nearest matrix in the symmetric solution set of the matrix Eq. (1) to a given matrix in the sense of the Frobenius norm, that is, finding $X$ such that

2 $$\mathop {\hbox{min} }\limits_{X} \frac{1}{2}\left\| {X - \tilde{X}} \right\|^{2} \quad subject\;to\;AXB = C , \quad \;X \in SR^{n \times n}$$

is called the matrix nearness problem. The matrix nearness problem is initially proposed in the processes of test or the recovery of linear systems due to incomplete dates or revising dates. A preliminary estimate $\tilde{X}$ of the unknown matrix $X$ can be obtained by the experimental observation values and the information of static distribution. The matrix nearness problem (2) with unknown matrix $X$ being symmetric, skew-symmetric and generalized reflexive were considered by Liao et al. (2007) (see also Peng et al. 2005), Huang et al. (2008) and Yuan et al. (2008), respectively. The approaches taken in these papers include the generalized singular value decomposition method and the matrix-form CGNE iteration method. In addition, there are many important results on the discussions of the matrix nearness problem associated with the other matrix equations, we refer the readers to (Chu and Golub 2005; Deng and Hu 2005; Higham 1988; Jin and Wei 2004; Konstaintinov et al. 2003; Penrose 1956) and references therein.

In this paper, we continue to consider the matrix nearness problem (2). By discussing the equivalent form of the matrix nearness problem (2), we derive some necessary and sufficient conditions for the matrix $X^{*}$ is a solution of the matrix nearness problem (2). Based on the idea of the alternating variable minimization with multiplier (AVMM) method (Bai and Tao 2015), we propose two iterative methods to compute the solution of the matrix nearness problem (2), and analyze the global convergence results of the proposed algorithms. Numerical comparisons with some existing methods are also given.

Throughout this paper the following notations are used. $R^{m \times n}$ and $SR^{n \times n}$ denote the set of $m \times n$ real matrices and the set of $n \times n$ real symmetric matrices. *I* denote the identity matrix with size implied by context. $A^{ + }$ denote the Moore–Penrose generalized inverse of the matrix $A$. Define the inner product in space $R^{m \times n}$ by $\left\langle {A,B} \right\rangle = tr(A^{T} B)$ for all $A,B \in R^{m \times n}$, then the associated norm is the Frobenius norm, and denoted by $\left\| A \right\|$.

## Iteration methods to solve the matrix nearness problem (2)

In this section we first give the equivalent constrained optimization problems of the matrix nearness problem (2), and discuss the properties of the solutions of these constrained optimization problems. Then we propose iteration methods to compute the solution of the equivalent constrained optimization problems, and hence to compute the solution of the matrix nearness problem (2). Finally, we prove some convergence theorems of the proposed algorithms.

Obviously, the matrix nearness problem (2) is equivalent to the following constrained optimization problem

3 $$\mathop {\hbox{min} }\limits_{X,Y} F(X,Y) = \frac{1}{2}\left\| {X - \tilde{X}} \right\|^{2} \quad \;subject\;to\;AX - Y = 0, \quad \;YB - C = 0, \quad \;X \in SR^{n \times n}$$

or

4 $$\mathop {\hbox{min} }\limits_{X,Y} F(X,Y) = \frac{1}{2}\left\| {X - \tilde{X}} \right\|^{2} \quad subject\;to\;\,XB - Y = 0, \quad \;AY - C = 0, \quad \;X \in SR^{n \times n}$$

**Theorem 1*** Matrix pair*$[X^{*} \vdots Y^{*} ]$* is a solution of the constrained optimization problem* (3)* if and only if exists matrices *$M^{*} \in R^{m \times n}$* and *$N^{*} \in R^{m \times p}$* such that the following equalities *(5–8)* hold*.

5 $$(X^{*} - \tilde{X} - A^{T} M^{*} ) + (X^{*} - \tilde{X} - A^{T} M^{*} )^{T} = 0 ,$$

6 $$M^{*} - N^{*} B^{T} = 0 ,$$

7 $$AX^{*} - Y^{*} = 0 ,$$

8 $$Y^{*} B - C = 0 .$$

*Proof* Assume that there exist matrices $M^{*}$ and $N^{*}$ such that the equalities (5–8) hold. Let

$$\bar{F}(X,Y) = F(X,Y) - \left\langle {M^{*} ,AX - Y} \right\rangle - \left\langle {N^{*} ,YB - C} \right\rangle.$$

Then, for any matrices $U \in SR^{n \times n}$ and $V \in R^{m \times n}$, we have

$$\begin{aligned} \bar{F}(X^{*}\,+\,U,Y^{*} + V) &= \tfrac{1}{2}\left\| {X^{*}\,+\,U - \tilde{X}} \right\|^{2} - \left\langle {M^{*} ,A(X^{*} + U)\,-\,(Y^{*} + V)} \right\rangle - \left\langle {N^{*} ,(Y^{*} + V)B - C} \right\rangle \\ &= \bar{F}(X^{*} ,Y^{*} ) + \tfrac{1}{2}\left\| U \right\|^{2} + \left\langle {U,X^{*} - \tilde{X}} \right\rangle - \left\langle {M^{*} ,AU - V} \right\rangle - \left\langle {N^{*} ,VB} \right\rangle \\&= \bar{F}(X^{*} ,Y^{*} ) + \tfrac{1}{2}\left\| U \right\|^{2} + \tfrac{1}{2}\left\langle {U,(X^{*} - \tilde{X} - A^{T} M^{*} ) + (X^{*} - \tilde{X} - A^{T} M^{*} )^{T} } \right\rangle + \left\langle {V,M^{*} - N^{*} B^{T} } \right\rangle\\ &= \bar{F}(X^{*} ,Y^{*} ) + \tfrac{1}{2}\left\| U \right\|^{2} \ge F(X^{*} ,Y^{*} ) \end{aligned}.$$

This implies that the matrix pair $[X^{*} \vdots Y^{*} ]$ is a global minimizer of the matrix function $\bar{F}(X,Y)$. Since $AX^{*} - Y^{*} = 0$, $Y^{*} B - C = 0$ and $\bar{F}(X,Y) \ge \bar{F}(X^{*} ,Y^{*} )$ hold for all $X \in SR^{n \times n}$ and $Y \in R^{m \times n}$, we have

$$F(X,Y) \ge F(X^{*} ,Y^{*} ) + \left\langle {M^{*} ,AX - Y} \right\rangle + \left\langle {N^{*} ,YB - C} \right\rangle.$$

Hence, $F(X,Y) \ge F(X^{*} ,Y^{*} )$ holds for all $X \in SR^{n \times n} ,Y \in R^{m \times n}$ with $AX - Y = 0$ and $YB - C = 0$. That is, the matrix pair $[X^{*} \vdots Y^{*} ]$ is a solution of the constrained optimization problem (3).

Conversely, if the matrix pair $[X^{*} \vdots Y^{*} ]$ is a global solution of the constrained optimization problem (3), then the matrix pair $[X^{*} \vdots Y^{*} ]$ certainly satisfies Karush–Kuhn–Tucker conditions of the constrained optimization problem (3). That is, there exist matrices $M^{*} \in R^{m \times n}$ and $N^{*} \in R^{m \times p}$ such that satisfy conditions (5–8). □

**Theorem 2*** Matrix pair *$[X^{*} \vdots Y^{*} ]$* is a solution of the constrained optimization problem* (4)* if and only if exists matrices*$M^{*} \in R^{n \times p}$* and *$N^{*} \in R^{m \times p}$* such that the following equalities *(9–12)* hold*.

9 $$(X^{*} - \tilde{X} - M^{*} B^{T} ) + (X^{*} - \tilde{X} - M^{*} B^{T} )^{T} = 0 ,$$

10 $$M^{*} - A^{T} N^{*} = 0 ,$$

11 $$X^{*} B - Y^{*} = 0 ,$$

12 $$AY^{*} - C = 0 .$$

*Proof* The proof is similar to Theorem 1 and is omitted here.□

Let

13 $$L_{\alpha ,\beta } (X,Y,M,N) = \frac{1}{2}\left\| {X - \tilde{X}} \right\|^{2} - \left\langle {M,AX - Y} \right\rangle - \left\langle {N,YB - C} \right\rangle + \frac{\alpha }{2}\left\| {AX - Y} \right\|^{2} + \frac{\beta }{2}\left\| {YB - C} \right\|^{2}$$

We propose an iteration method to solve the constrained optimization (3), and hence to solve the matrix nearness problem (2) as follows.

## **Algorithm 1**

Step 1. Input the matrices $A,B,C,\tilde{X}$ and the tolerance $\varepsilon > 0$. Choose the initial matrices $Y_{0} ,M_{0} ,N_{0}$ and the parameters $\alpha ,\beta > 0$. Set $k \leftarrow 0$.
Step 2. Exit if a stopping criterion has been met.
Step 3. Compute
  14 $$X_{k + 1} = \arg \mathop {\hbox{min} }\limits_{{X \in SR^{n \times n} }} L_{\alpha ,\beta } (X,Y_{k} ,M_{k} ,N_{k} ) ,$$
  15 $$Y_{k + 1} = \arg \mathop {\hbox{min} }\limits_{{Y \in R^{m \times n} }} L_{\alpha ,\beta } (X_{k + 1} ,Y,M_{k} ,N_{k} ) ,$$
  16 $$M_{k + 1} = M_{k} - \alpha (AX_{k + 1} - Y_{k + 1} ) ,$$
  17 $$N_{k + 1} = N_{k} - \beta (Y_{k + 1} B - C),$$
  Set $k \leftarrow k + 1$ and go to Step 2.
Step 4. Let
  18 $$\tilde{L}_{\alpha ,\beta } (X,Y,M,N) = \frac{1}{2}\left\| {X - \tilde{X}} \right\|^{2}\,-\,\left\langle {M,XB - Y} \right\rangle - \left\langle {N,AY - C} \right\rangle \,+ \, \frac{\alpha }{2}\left\| {XB - Y} \right\|^{2} \,+\, \frac{\beta }{2}\left\| {AY - C} \right\|^{2} .$$

We similarly propose an iteration method to solve the constrained optimization (4), and hence to solve the matrix nearness problem (2) as follows.

### **Algorithm 2**

Step 1. Input the matrices $A,B,C,\tilde{X}$ and the tolerance $\varepsilon > 0$. Choose the initial matrices $Y_{0} ,M_{0} ,N_{0}$ and the parameters $\alpha ,\beta > 0$. Set $k \leftarrow 0$.
Step 2. Exit if a stopping criterion has been met.
Step 3. Compute
  19 $$X_{k + 1} = \arg \mathop {\hbox{min} }\limits_{{X \in SR^{n \times n} }} \tilde{L}_{\alpha ,\beta } (X,Y_{k} ,M_{k} ,N_{k} ) ,$$
  20 $$Y_{k + 1} = \arg \mathop {\hbox{min} }\limits_{{Y \in R^{m \times n} }} \tilde{L}_{\alpha ,\beta } (X_{k + 1} ,Y,M_{k} ,N_{k} ) ,$$
  21 $$M_{k + 1} = M_{k} - \alpha (X_{k + 1} B - Y_{k + 1} ) ,$$
  22 $$N_{k + 1} = N_{k} - \beta (AY_{k + 1} - C),$$
Step 4. Set $k \leftarrow k + 1$ and go to Step 2.

For the Algorithm 1 and 2, the most of time consumption is compute $X_{k + 1}$ and $Y_{k + 1}$. Below, we discuss how to compute $X_{k + 1}$ and $Y_{k + 1}$. Firstly, $X_{k + 1}$ in (14) can be expressed as

$$\begin{aligned} X_{k + 1} = \arg \mathop {\hbox{min} }\limits_{{X \in SR^{n \times n} }} \frac{1}{2}\left\| {X - \tilde{X}} \right\|^{2} - \left\langle {M_{k} ,AX - Y_{k} } \right\rangle + \frac{\alpha }{2}\left\| {AX - Y_{k} } \right\|^{2} \\ & = \arg \mathop {{\rm{min}}}\limits_{X \in S{R^{n \times n}}} \frac{1}{2}{\left\| {X - \tilde X} \right\|^2} + \frac{\alpha }{2}{\left\| {AX - \left( {{Y_k} + \frac{1}{\alpha }{M_k}} \right)} \right\|^2} \\ &= \arg \mathop {\hbox{min} }\limits_{{X \in SR^{n \times n} }} \frac{1}{2}\left\| {\left[ {\begin{array}{*{20}c} {\sqrt \alpha A} \\ I \\ \end{array} } \right]X - \left[ {\begin{array}{*{20}c} {\sqrt \alpha Y_{k} + M_{k} /\sqrt \alpha } \\ {\tilde{X}} \\ \end{array} } \right]} \right\|^{2} \end{aligned}$$

23 $$= \arg \mathop {\hbox{min} }\limits_{{X \in SR^{n \times n} }} \frac{1}{2}\left\| {SX - T} \right\|^{2}$$

where $$S = \left[ {\begin{array}{*{20}c} {\sqrt \alpha A} \\ I \\ \end{array} } \right] \in R^{(m + n) \times n}$$, $$T = \left[ {\begin{array}{*{20}c} {\sqrt \alpha Y_{k} + M_{k} /\sqrt \alpha } \\ {\tilde{X}} \\ \end{array} } \right] \in R^{(m + n) \times n}$$. Analogously, $X_{k + 1}$ in (19) can be expressed as

24 $$X_{k + 1} = \arg \mathop {\hbox{min} }\limits_{{X \in SR^{n \times n} }} \frac{1}{2}\left\| {XS - T} \right\|^{2}$$

where $$S = \left[ {I,\sqrt \alpha B} \right] \in R^{n \times (n + p)}$$, $$T = \left[ {\tilde{X},\sqrt \alpha Y_{k} + M_{k} /\sqrt \alpha } \right] \in R^{n \times (n + p)}$$.

To solve the problems (23) and (24), we give the following Lemma 1.

**Lemma 1** (Sun 1988) *Given matrices *$$B \in R^{n \times n}$$* and , *$$\Upsigma = diag(\sigma_{1} ,\sigma_{2} , \ldots \sigma_{n} )$$*, then the problem *$$\sigma_{i} > 0\,(i = 1, \ldots n)\,\,$$$$\left\| {X\Upsigma - B} \right\|^{2} = \hbox{min}$$* have a unique least squares symmetric solution with the following expression*

$$X = \Upphi \circ (B\Upsigma + \Upsigma B^{T} )$$

*where*$\Upphi_{ij} = \frac{1}{{\sigma_{i}^{2} + \sigma_{j}^{2} }}$, $\Upphi = (\Upphi_{ij} ) \in R^{n \times n},$* and*$A \circ B = (a_{ij} b_{ij} )$* denotes the Hadamard product.*

Noting that the matrix $S$ in (23) is full column rank, the singular value decomposition (SVD) of the matrix $S$ can be expressed as

$$S = U\left( {\begin{array}{*{20}c} \Upsigma \\ 0 \\ \end{array} } \right)V^{T}$$

where $\Upsigma = diag(\sigma_{1} , \ldots ,\sigma_{n} )$, $\sigma_{i} > 0$, and $U = (U_{1} ,U_{2} ) \in R^{(m + n) \times (m + n)}$, $V \in R^{n \times n}$ are orthogonal matrices, $U_{1} \in R^{(m + n) \times n}$. Hence, $X_{k + 1}$ in (23) can be expressed as

$$\begin{aligned} X_{k + 1}& = \arg \mathop {\hbox{min} }\limits_{{X \in SR^{n \times n} }} \frac{1}{2}\left\| {U\left( {\begin{array}{*{20}c} \Upsigma \\ 0 \\ \end{array} } \right)V^{T} X - T} \right\|^{2} \\ & = \arg \mathop {\hbox{min} }\limits_{{X \in SR^{n \times n} }} \frac{1}{2}\left\| {\left( {\begin{array}{*{20}c} \Upsigma \\ 0 \\ \end{array} } \right)V^{T} XV - U^{T} TV} \right\|^{2} \\ & = \arg \mathop {\hbox{min} }\limits_{{X \in SR^{n \times n} }} \frac{1}{2}\left\| {\left( {\begin{array}{*{20}c} \Upsigma \\ 0 \\ \end{array} } \right)V^{T} XV - \left( {\begin{array}{*{20}c} {U_{1}^{T} } \\ {U_{2}^{T} } \\ \end{array} } \right)TV} \right\|^{2} \\ &= \arg \mathop {\hbox{min} }\limits_{{X \in SR^{n \times n} }} \frac{1}{2}\left\| {\Upsigma V^{T} XV - U_{1}^{T} TV} \right\|^{2}\end{aligned}$$

Let $\tilde{T} = U_{1}^{T} TV$, we have by Lemma 1 that $X_{k + 1}$ in (23) can be expressed as

$$X_{k + 1} = V(\Upphi \circ (\Upsigma \tilde{T} + \tilde{T}^{T} \Upsigma ))V^{T}$$

Analogously, the matrix $S$ in (24) is full row rank, and the SVD of $S$ can be expressed as

$$S = P(\Upsigma ,0)Q^{T}$$

where $\Upsigma = diag(\sigma_{1} , \ldots ,\sigma_{n} )$, $\sigma_{i} > 0$, and $P \in R^{n \times n}$, $Q = (Q_{1} ,Q_{2} ) \in R^{(n + p) \times (n + p)}$ are the orthogonal matrices, $Q_{1} \in R^{(n + p) \times n}$, and $X_{k + 1}$ in (24) can be expressed as

$$X_{k + 1} = P(\Upphi \circ (\Upsigma \tilde{T} + \tilde{T}^{T} \Upsigma ))P^{T}$$

where $$\tilde{T} = P^{T} TQ_{1}$$.

Then, we change our attention to compute $Y_{k + 1}$. By simply changing, $Y_{k + 1}$ in (15) can be expressed as

$$\begin{aligned}Y_{k + 1} = \arg \mathop {\hbox{min} }\limits_{{Y \in R^{m \times n} }} \frac{1}{2}\left\| {Y\left( {\sqrt \alpha I,\sqrt \beta B} \right) - \left( {\sqrt \alpha AX_{k + 1} - M_{k} /\sqrt \alpha ,\sqrt \beta C + N_{k} /\sqrt \beta } \right)} \right\|^{2} \\ & = \left( {\sqrt \alpha AX_{k + 1} - M_{k} /\sqrt \alpha ,\sqrt \beta C + N_{k} /\sqrt \beta } \right)\left( {\sqrt \alpha I,\sqrt \beta B} \right)^{ + } \end{aligned}$$

and $Y_{k + 1}$ in (20) can be expressed as

$$\begin{aligned} Y_{k + 1} = \arg \mathop {\hbox{min} }\limits_{{Y \in R^{n \times p} }} \frac{1}{2}\left\| {\left( {\begin{array}{*{20}c} {\sqrt \alpha I} \\ {\sqrt \beta A} \\ \end{array} } \right)Y - \left( {\begin{array}{*{20}c} {\sqrt \alpha X_{k + 1} B - M_{k} /\sqrt \alpha } \\ {\sqrt \beta C + N_{k} /\beta } \\ \end{array} } \right)} \right\|^{2} \\ &= \left( {\begin{array}{*{20}c} {\sqrt \alpha I} \\ {\sqrt \beta A} \\ \end{array} } \right)^{ + } \left( {\begin{array}{*{20}c} {\sqrt \alpha X_{k + 1} B - M_{k} /\sqrt \alpha } \\ {\sqrt \beta C + N_{k + 1} /\sqrt \beta } \\ \end{array} } \right) \end{aligned}$$

Next, we discuss the global convergence of Algorithm 1 and 2. Note that Algorithm 2 is similar to Algorithm 1, we only discuss the convergence of Algorithm 1.

**Theorem 3*** Let *$(X^{*} ,Y^{*} ,M^{*} ,N^{*} )$* be a saddle point for the Lagrange function*

$$L(X,Y,M,N) = \frac{1}{2}\left\| {X - \tilde{X}} \right\|^{2} - \left\langle {M,AX - Y} \right\rangle - \left\langle {N,YB - C} \right\rangle$$

*of the constrained optimization problem *(3)*, that is, the matrices*$X^{*} ,Y^{*} ,M^{*} ,N^{*}$*satisfy conditions *(5–8)*. Define*

$$S_{k + 1} = AX_{k + 1} - Y_{k + 1},\,\, T_{k + 1} = Y_{k + 1} B - C, \,\, \mu_{k} = \alpha \left\| {Y_{k} - Y^{*} } \right\|^{2} + \frac{1}{\alpha }\left\| {M_{k} - M^{*} } \right\|^{2} + \frac{1}{\beta }\left\| {N_{k} - N^{*} } \right\|^{2},$$

*then, the following inequality holds*

25 $$\mu_{k + 1} \le \mu_{k} - \beta \left\| {T_{k + 1} } \right\|^{2} - \alpha \left\| {S_{k + 1} + Y_{k + 1} - Y_{k} } \right\|^{2} .$$

*Proof* Since $(X^{*} ,Y^{*} ,M^{*} ,N^{*} )$ is a saddle point, we have by the saddle point theorem (Bjorck 2006) that

$$L(X^{*} ,Y^{*} ,M,N) \le L(X^{*} ,Y^{*} ,M^{*} ,N^{*} ) \le L(X,Y,M^{*} ,N^{*} ),$$

for all $X,Y,M,N$, where $L(X,Y,M,N) = \frac{1}{2}\left\| {X - \tilde{X}} \right\|^{2} - \left\langle {M,AX - Y} \right\rangle - \left\langle {N,YB - C} \right\rangle$, which called the Lagrange function of the constrained optimization problem (3). Hence we have

$$L(X^{*} ,Y^{*} ,M^{*} ,N^{*} ) \le L(X_{k + 1} ,Y_{k + 1} ,M^{*} ,N^{*} )$$

Noting that $AX^{*} - Y^{*} = 0 , \, Y^{*} B - C = 0$, $S_{k + 1} = AX_{k + 1} - Y_{k + 1}$ and $T_{k + 1} = Y_{k + 1} B - C$, we know that the following inequality holds

26 $$\frac{1}{2}\left\| {X^{*} - \tilde{X}} \right\|^{2} - \frac{1}{2}\left\| {X_{k + 1} - \tilde{X}} \right\|^{2} \le - \left\langle {M^{*} ,S{}_{k + 1}} \right\rangle - \left\langle {N^{*} ,T_{k + 1} } \right\rangle ,$$

Since $X_{k + 1}$ minimize the matrix function $L_{\alpha ,\beta } (X,Y_{k} ,M_{k} ,N_{k} )$, we have

27 $$\begin{aligned} 0 &= \left[ {X_{k + 1} - \tilde{X} - A^{T} M_{k} + \alpha A^{T} (AX_{k + 1} - Y_{k} )} \right] + \left[ {X_{k + 1} - \tilde{X} - A^{T} M_{k} + \alpha A^{T} (AX_{k + 1} - Y_{k} )} \right]^{T} \\ &= \left[ {X_{k + 1} - \tilde{X} - A^{T} M_{k + 1} - \alpha A^{T} (Y_{k} - Y_{k + 1} )} \right] + \left[ {X_{k + 1} - \tilde{X} - A^{T} M_{k + 1} - \alpha A^{T} (Y_{k} - Y_{k + 1} )} \right]^{T} , \end{aligned}$$

where the first equality is the first-order optimality condition of the problem (14), and the second equality is followed by Algorithm 1. This implies that

$$X_{k + 1} = \arg \mathop {\hbox{min} }\limits_{{X \in SR^{n \times n} }} \frac{1}{2}\left\| {X - \tilde{X}} \right\|^{2} - \left\langle {M_{k + 1} - \alpha Y_{k + 1} + \alpha Y_{k} ,AX} \right\rangle$$

Hence, we have

28 $$\begin{aligned}&\frac{1}{2}\left\| {X_{k + 1} - \tilde{X}} \right\|^{2} - \left\langle {M_{k + 1} - \alpha Y_{k + 1} + \alpha Y_{k} ,AX_{k + 1} } \right\rangle \\ &\quad \le \frac{1}{2}\left\| {X^{*} - \tilde{X}} \right\|^{2} - \left\langle {M_{k + 1} - \alpha Y_{k + 1} + \alpha Y_{k} ,AX^{*} } \right\rangle . \end{aligned}$$

Since $Y_{k + 1}$ minimize $L_{\alpha ,\beta } (X_{k + 1} ,Y,M_{k} ,N_{k} )$, we have

29 $$\begin{aligned}0 &= M_{k} - N_{k} B^{T} - \alpha (AX_{k + 1} - Y_{k + 1} ) + \beta (Y_{k + 1} B - C)B^{T}\\ & = M_{k + 1} - N_{k + 1} B^{T} , \end{aligned}$$

where the first equality is the first-order optimality condition of the problem (15), and the second equality is followed by Algorithm 1. This implies that

$$Y_{k + 1} = \arg \mathop {\hbox{min} }\limits_{{Y \in R^{m \times n} }} \left\langle {M_{k + 1} - N_{k + 1} B,Y} \right\rangle$$

Hence, we have

30 $$\left\langle {M_{k + 1} - N_{k + 1} B^{T} ,Y_{{{\text{k}} + 1}} } \right\rangle \le \left\langle {M_{k + 1} - N_{k + 1} B^{T} ,Y^{*} } \right\rangle$$

Adding the inequalities (28) and (30), and using $AX^{*} - Y^{*} = 0$, $Y^{*} B - C = 0$, we know that the following inequality holds

31 $$\begin{aligned} &\frac{1}{2}\left\| {X_{k + 1} - \tilde{X}} \right\|^{2} - \frac{1}{2}\left\| {X^{*} - \tilde{X}} \right\|^{2}\\ &\quad \le \left\langle {M_{k + 1} ,S{}_{k + 1}} \right\rangle + \left\langle {N_{k + 1} ,T_{k + 1} } \right\rangle - \alpha \left\langle {Y_{k + 1} - Y_{k} ,S_{k + 1} + Y_{k + 1} - Y^{*} } \right\rangle , \end{aligned}$$

Adding the inequalities (26) and (31), we have

32 $$\left\langle {M^{*} - M_{k + 1} ,S_{k + 1} } \right\rangle + \left\langle {N^{*} - N_{k + 1} ,T_{k + 1} } \right\rangle + \alpha \left\langle {Y_{k + 1} - Y_{k} ,S_{k + 1} + Y_{k + 1} - Y^{*} } \right\rangle \le 0.$$

Noting that

$$\begin{aligned} &2\left\langle {M^{*} - M_{k + 1} ,S_{k + 1} } \right\rangle + 2\left\langle {N^{*} - N_{k + 1} ,T_{k + 1} } \right\rangle \\ &\quad= 2\left\langle {M^{*} - M_{k} ,S_{k + 1} } \right\rangle + 2\alpha \left\| {S_{k + 1} } \right\|^{2} + 2\left\langle {N^{*} - N_{k} ,T_{k + 1} } \right\rangle + 2\beta \left\| {T_{k + 1} } \right\|^{2}\\ &\quad= \frac{2}{\alpha }\left\langle {M^{*} - M_{k} ,M_{k} - M_{k + 1} } \right\rangle + \frac{1}{\alpha }\left\| {M_{k} - M_{k + 1} } \right\|^{2} + \alpha \left\| {S_{k + 1} } \right\|^{2}\\ &\qquad + \frac{2}{\beta }\left\langle {N^{*} - N_{k} ,N_{k} - N_{k + 1} } \right\rangle + \frac{1}{\beta }\left\| {N_{k} - N_{k + 1} } \right\|^{2} + \beta \left\| {T_{k + 1} } \right\|^{2}\\ &\quad = \frac{1}{\alpha }\left( {\left\| {M_{k + 1} - M^{*} } \right\|^{2} - \left\| {M_{k} - M^{*} } \right\|^{2} } \right) + \alpha \left\| {S_{k + 1} } \right\|^{2} \\ &\qquad + \frac{1}{\beta }\left( {\left\| {N_{k + 1} - N^{*} } \right\|^{2} - \left\| {N_{k} - N^{*} } \right\|^{2} } \right) + \beta \left\| {T_{k + 1} } \right\|^{2}\end{aligned}$$

and

$$\begin{aligned}\alpha \left\| {S_{k + 1} } \right\|^{2} + 2\alpha \left\langle {Y_{k + 1} - Y_{k} ,S_{k + 1} + Y_{k + 1} - Y^{*} } \right\rangle\\ &\quad= \alpha \left\| {S_{k + 1} + Y_{k + 1} - Y_{k} } \right\|^{2} + \alpha \left\| {Y_{k + 1} - Y_{k} } \right\|^{2} + 2\alpha \left\langle {Y_{k + 1} - Y_{k} ,Y_{k} - Y^{*} } \right\rangle \\ & \quad = \alpha \left\| {S_{k + 1} + Y_{k + 1} - Y_{k} } \right\|^{2} + \alpha \left( {\left\| {Y_{k + 1} - Y^{*} } \right\|^{2} - \left\| {Y_{k} - Y^{*} } \right\|^{2} } \right), \end{aligned}$$

We have by inequality (32) and the definition of $\mu_{k}$ that

$$\mu_{k + 1} \le \mu_{k} - \beta \left\| {T_{k + 1} } \right\|^{2} - \alpha \left\| {S_{k + 1} + Y_{k + 1} - Y_{k} } \right\|^{2}$$

which means that the inequality (25) holds. The proof is completed. □

Theorem 3 implies that the sequence $\left\{ {\mu_{k} } \right\}$ is a nonnegative monotone decreasing with low bounded. Hence, the limit of the sequence $\left\{ {\mu_{k} } \right\}$ exists which implies that the limit of the sequences $\left\{ {Y_{k} } \right\}$, $\left\{ {M_{k} } \right\}$, $\left\{ {N_{k} } \right\}$ exist, and $S_{k + 1} + Y_{k + 1} - Y_{k} = AX_{k + 1} - Y_{k} \to 0$ and $T_{k + 1} = Y_{k + 1} B - C \to 0$ as $k \to \infty$. Futhermore, $S_{k + 1} + Y_{k + 1} - Y_{k} = AX_{k + 1} - Y_{k} \to 0$ as $k \to \infty$ implies that the limit of the sequence $\left\{ {X_{k} } \right\}$ exists. Assume that $X_{k} \to X^{*}$, $Y_{k} \to Y^{*}$, $M_{k} \to M^{*}$, $N_{k} \to N^{*}$ as $k \to \infty$, then (9) and (10) are hold by taking limit, respectively, the Eqs. (27) and (29), and (11) and (12) are hold by $S_{k + 1} + Y_{k + 1} - Y_{k} = AX_{k + 1} - Y_{k} \to 0$ and $T_{k + 1} = Y_{k + 1} B - C \to 0$ as $k \to \infty$. Hence, we have by Theorem 1 that the matrix pair $[X^{*} \vdots Y^{*} ]$ is a solution of the constrained optimization problem (3), and hence is a solution of the matrix nearness problem (2). In addition, Note that the subjective function of the constrained optimization problem (3) is a strictly convex functions and the constrained set $\Upomega = \left\{ {\left[ {X \vdots Y} \right]\left| {AX - Y = 0,YB - C = 0,X \in SR^{n \times n} } \right.} \right\}$ is closed and convex, we know that matrix pair $[X^{*} \vdots Y^{*} ]$ is the unique solution of problem (3). Hence the sequence $\left\{ {X_{k} } \right\}$ generated by Algorithm 1 converges to the unique solution of the matrix nearness problem (2). These results can be described as the following Theorem 4.

**Theorem 4*** Assume that *$$\left\{ {X_{k} } \right\}$$* is a sequence generated by Algorithm 1 with any initial matrices *$$Y_{0} ,M_{0} ,N_{0}$$* and the parameters, then the sequence *$$\alpha ,\beta > 0$$$$\left\{ {X_{k} } \right\}$$* converges to a solution of the matrix nearness problem* (2).

## Numerical experiments

In this section, we compare Algorithm 1 and 2 with existing two methods proposed in (Peng et al. 2005; Peng 2010), denoted, respectively, by CG and LSQR. Our computational experiments were performed on an IBM ThinkPad T410 with 2.5 GHz and 3.0 RAM. All tests were performed in MATLAB 7.1 with a 64-bit Windows 7 operating system.

In the implementation of Algorithm 1 and 2, we take parameters $\alpha = \beta = 10$. The initial matrices $Y_{0} ,M_{0} ,N_{0}$ in Algorithm 1 and 2, and $X_{0}$ in Algorithm CG and LSQR are chosen as zeros matrices. All of algorithms, the small tolerance $\varepsilon = 10^{ - 8}$ and the termination criterion are chosen as $\left\| {AX_{k} B -C} \right\| \le \varepsilon$. In addition, the maximum iterations numbers of the three methods is limited within 20000.

For the matrix nearness problem (2), the matrices $A,B,\tilde{X}$ and $C$ are given as follows (in MATLAB style): $A = randn(m,n)$, $B = randn(n,p)$, $\tilde{X} = randn(n,n)$, $C = AX_{0} B$ with $X_{0} = H + H^{T}$ and $H = randn(n,n)$. Here the matrix *C* is chosen in this way to guarantee that the matrix nearness problem (2) is solvable.

In Table 1, we report the iteration CPU time (‘CPU’) and the iteration numbers (‘IT’) based on their average values of 10 repeated tests with randomly generated matrices *A, B* and *C* for each problem size in each tests.

**Table 1:** Iteration time (seconds) and iteration numbers for four methods

m, n, p	Algorithm 1		Algorithm 2		Algorithm CG		Algorithm LSQR	
	IT	CPU	IT	CPU	IT	CPU	IT	CPU
20, 20, 20	2807	1.0407	1992	0.9338	941	0.1603	816	0.1181
40, 40, 40	6709	60.3186	5085	52.1359	3445	25.3471	2925	21.6702
60, 60, 60	8109	74.2018	7882	67.0493	7203	45.1428	5987	43.2527
80, 80, 80	8246	88.6394	5503	73.6081	10,974	88.6511	9773	83.7413
100, 80, 80	5795	67.6304	2081	23.6263	6681	59.9316	5636	54.6361
200, 80, 80	9445	130.5067	2503	12.2904	4721	60.9609	4913	55.7173
400, 80, 60	13,756	252.4951	2234	0.2925	1885	22.5552	1518	20.8768
80, 80, 100	2843	46.9736	6082	82.7429	7393	66.6155	6228	59.0029
80, 80, 200	728	8.8492	7324	101.4176	4768	62.8689	4855	54.1167
60, 80, 400	25	0.4173	14,009	258.8992	2245	40.7279	1786	22.5928
100, 80, 100	332	3.9936	352	4.1774	2011	18.5992	1604	16.1305
200, 80, 200	39	0.7021	38	0.6241	531	7.8664	459	6.9108
200, 80, 400	21	0.5096	41	0.9568	299	11.5416	348	7.3581
400, 80, 200	41	1.0451	22	0.4941	415	8.7829	361	7.8676

Based on the tests reported in Table 1 and many other performed unreported tests which show similar patterns, we have the following results: when $m,p \gg n$, Algorithm 1 and 2 are more effective than Algorithms CG and LSQR. But when $m \approx p \approx n$, Algorithm CG and LSQR are relatively more effective than Algorithms 1 and 2. When $p < m$ and $m \gg n$, Algorithm 2 is the most effective, and Algorithm 1 is the most effective when $m < p$ and $p \gg n$.

## Conclusions

In this paper, we have considered the matrix nearness problem (2), i.e., finding the matrix nearness solution $X^{*}$ of matrix equation $AXB = C$ to a given matrix $\tilde{X}$. By discussing equivalent form of the considered problem, we have derived some necessary and sufficient conditions for the matrix $X^{*}$ is a solution of the considered problem. Based on the idea of the alternating variable minimization with multiplier method, we have proposed two iterative methods to compute the solution of the considered problem, and have analyzed global convergence results of the proposed algorithms. Numerical results illustrate proposed methods are more effective than existing two methods proposed in Peng et al. (2005) and Peng (2010).
